# “*Candidatus* anaplasma camelii” in one-humped camels (*Camelus dromedarius*) in Morocco: a novel and emerging *anaplasma* species?

**DOI:** 10.1186/s40249-016-0216-8

**Published:** 2017-02-05

**Authors:** Hicham Ait Lbacha, Zaid Zouagui, Said Alali, Abdelkbir Rhalem, Elisabeth Petit, Marie Julie Ducrotoy, Henri-Jean Boulouis, Renaud Maillard

**Affiliations:** 1 Institut Agronomique et Vétérinaire Hassan II, Rabat, Morocco; 2 Département de médecine, chirurgie et reproduction, Madinat Al Irfane, Rabat Instituts, IAV Hassan II, BP 6202, Rabat, 10101 Morocco; 3 Anses, ENVA, INRA, BIPAR, Maisons-Alfort, France; 4 Division of Infection and Pathway Medicine, School of Biomedical Sciences, College of Medicine and Veterinary Medicine, The University of Edinburgh, Edinburgh, UK; 5 Ceva Santé Animale, Libourne, France; 6 INP-ENVT, Toulouse, France

**Keywords:** *Anaplasma camelii*, Tick-borne, One-humped camel (*Camelus dromedarius*), *groEL*, Phylogeny, Morocco

## Abstract

**Background:**

There has been a growing interest in camel anaplasmosis due to its recent emergence in this reservoir species and concerns for its zoonotic potential. The epidemiology of anaplasmosis in camels therefore remains poorly understood mostly because camels belong to marginalised poor and often transhumant populations whose interests are largely neglected. Most studies of anaplasmosis in camels have relied on microscopy and serology for diagnosis and only three studies, undertaken in Tunisia, Saudia Arabia and China, have used molecular diagnostics. The present work characterises Anaplasmataceae strains circulating in the *Camelus dromedarius* reservoir in Morocco using PCR.

**Methods:**

Camels (*n* = 106) were randomly sampled from 6 regions representing different agro-ecological areas in southern Morocco. Whole blood was collected and screened using PCR methods targeting the gene *groEL*. Anaplasmataceae strains were characterised by sequence analysis of the gene *groEL*.

**Results:**

A total of 39.62% (42/106) camels screened were positive for Anaplasmataceae spp. GenBank BLAST analysis of five positive sequenced samples revealed that all strains were 100% identical to “*Candidatus* Anaplasma camelii”. Phylogenetic investigation and genetic characterisation of the aligned segment (650 bp) of the gene *groEL* confirmed high similarity with *A. platys*.

**Conclusion:**

This study demonstrates the circulation of a previously unidentified species of the genus *Anaplasma* in Morocco which is genetically close to the agent causing canine anaplasmosis but whose main reservoir is thought to be *Camelus dromedarius*.

**Trial registration number:**

This study is not a clinical trial and therefore a trial registration number does not apply.

**Electronic supplementary material:**

The online version of this article (doi:10.1186/s40249-016-0216-8) contains supplementary material, which is available to authorized users.

## Multilingual abstracts

Please see Additional file 1 translations of the abstract into the six official working languages of the United Nations.

## Background

Tick-borne diseases, especially those caused by Rickettsiae, are a major source of economic burden for livestock keepers due to their impact on productivity in ruminant hosts and that several pathogens in this group are also zoonotic. Anaplasmosis is tick borne and caused by gram negative, obligate intracellular bacteria of the genus *Anaplasma* [1]. The epidemiology of anaplasmosis is complex due to the diversity of *Anaplasma* species that cause the condition, the wide host range and the role of a vector in its transmission.

The *Anaplasma* genus includes, but is not limited to, the following species: (1) *A. marginale*, (2) *A. centrale, (3) A. ovis, (4) A. bovis, (5) A. platys, and (6) A. phagocytophilum.* (1) *A. marginale* is the aetiological agent of bovine intra-erythrocytic anaplasmosis [2]. Infection occurs through the bite of a tick carrying the bacteria [3, 4]. Hard ticks, including *Rhipicephalus* spp., *Boophilus* spp., *Dermacentor* spp. and *Ixodes ricinus* are the main source of transmission, although other sources of biological and mechanical transmission have been reported [5, 6]. Post infection, the incubation lasts for 7 to 60 days after which if parasitaemia of red blood cells exceeds the 15% threshold, clinical signs appear [7, 8]. The severity of signs observed during the clinical phase varies depending on strain virulence and immune status of infected cattle. In general, infected cattle present with anaemia, pyrexia, lethargy, weight loss, milk drop in lactating females and occasionally abortion for in-calf cows. Death may occur in the absence of chemotherapy and veterinary care [7, 9]. (2) *A. centrale* preferentially infects cattle and is used as a live vaccine against *A. marginale* in cattle in Australia, South Africa and South America because of its lower virulence and good cross immunity [10]. Small ruminants are preferentially infected by *A. ovis* (3) and prevalence has been reported to be high in several countries [11–13] with considerable economic impact [13]. Clinical cases usually present in stressed, immune-depressed sheep and goats or in cases of co-infection with clinical signs similar to those observed for *A. marginale* infected cattle [14, 15]. *A. ovis* transmission to small ruminants occurs through tick bites as described for cattle, although *Rhipicephalus* spp. play a greater role [14].

In addition to intra-erythrocytic *Anaplasma* species, the genus also includes *A. bovis* (4), which causes intra-monocytic anaplasmosis, a sub-clinical or benign clinical form of the disease [16]. Other species include *A. platys* (5), which has a tropism for platelets in dogs and causes canine cyclic thrombocytopenia [17] and *A. phagocytophilum* (6) which causes tick-borne fever (TBF) in domestic ruminants [18], granulocytic anaplasmosis (GA) in humans [19], Equine GA in horses [20], canine GA in dogs [21] and feline GA in cats [22]. Like *A. phagocytophilum, A. ovis* has been found on rare occasions to be zoonotic [23, 24].

Despite the limited number of studies undertaken on anaplasmosis in camels, evidence to date would suggest that one-humped camels (*Camelus dromedarius*) are not a preferential host for the *Anaplasma.* The only *Anaplasma* species found in this camel are genetically related to *A. platys* [25–27]*.* BenSaid et al. (2014) [28] reported *A. phagocytophilum* seropostive camels in Tunisia but this serological diagnosis was not confirmed by molecular methods.

During the last three years, an outbreak of undiagnosed disease in camels causing clinical signs of dependant oedema, anorexia, respiratory distress and sudden death was reported in the southern regions of Morocco by livestock keepers and veterinary services. The presentation of this undiagnosed illness was similar to the clinical signs observed in cattle acutely infected with *A. phagocytophilum* and given the practice of trans-boundary transhumance across the Sahara of North Africa it was thought likely that camel anaplasmosis would be present in Morocco. The present study investigates and characterises Anaplasmataceae spp. infection in *Camelus dromedarius* in Morocco using molecular tools.

## Methods

### Region and study population

**Fig. 1:**
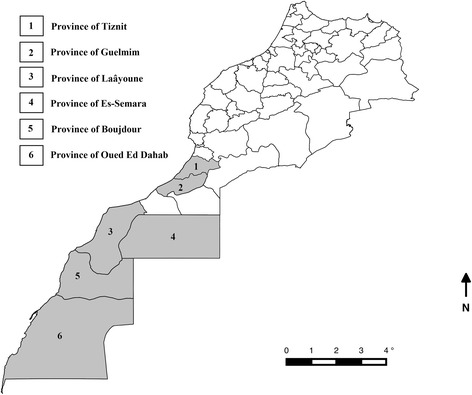
Map of Moroccan regions sampled

**Fig. 2:**
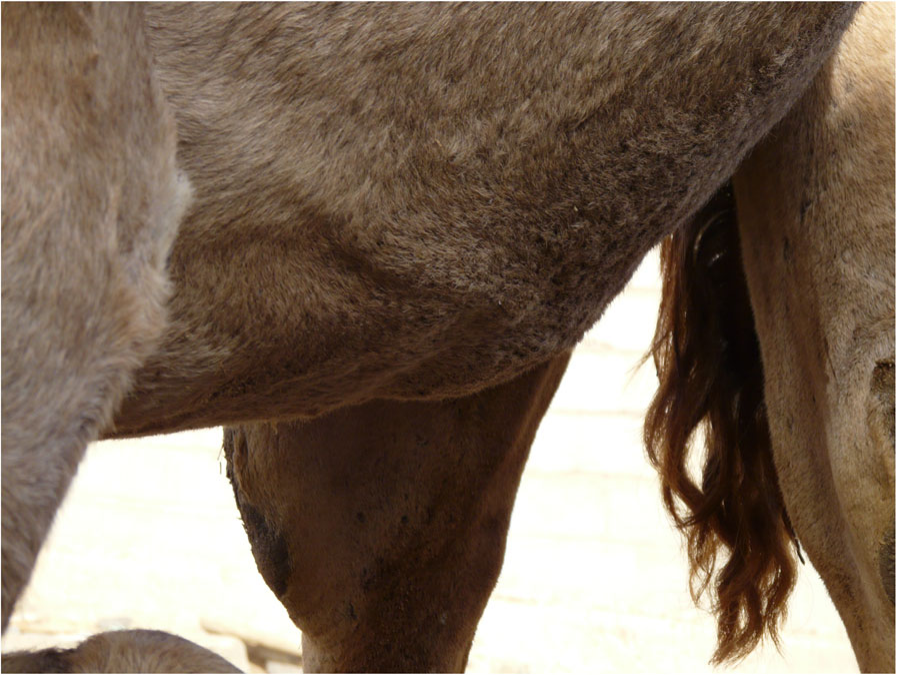
Dependent oedema in the region of the sternum and xiphoid in a 6 years old female camel

A cross-sectional survey was undertaken between December 2013 and April 2015 with camel herds were purposefully selected based on owner willingness to participate in the study. Sampling was conducted across 37 sites in six regions of southern Morocco including areas where the outbreak of undiagnosed disease was reported (Fig. 1). 106 camels were sampled in total. At the herd level, a sub-sample of camels was randomly tested. Four of the camels sampled showed signs of dependant oedema at the time of sampling (Fig. 2). Whole blood was collected from the jugular vein using EDTA vacutainers® and was subsequently aliquoted and stored at −20 °C until further analysis. Ticks were collected from camels and were identified using standard keys [29].

### DNA extraction

DNA was extracted from camel whole blood using the kit NucleoSpin® Blood Quick Pure (Macherey-Nagel, Düren, Germany) according to manufacturer instructions. DNA was stored at −20 °C until amplification.

### Polymerase chain reaction

All extracted DNA was amplified targeting the ‘heat-shock operon’ ‘*groEL*’ using Anaplasmataceae-specific PCR primers AnaplatF2 5’-GCGTAGTCCGATTCTCCAGT-3’ and AnaGro712R 5’-CCGCGATCAAACTGCATACC-3’ [25, 30]. A final PCR mix volume of 25 μl was prepared by adding 1.5 μl of each primer, 12.5 μl of Taq DNA Promega GoTaq® Hot Start Colorless Master Mix (Promega corporation, Madison,WI, USA), 4.5 μl DNA-free water and 5 μl of DNA to amplify. The thermocycler Eppendorf Mastercycler® (Eppendorf, Hamburg, Germany) was programmed for an initial denaturation at 95 °C during 8 min, followed by 35 cycles of denaturation at 94 °C during 1 min, hybridisation at 59 °C during 40 s and elongation at 72 °C during 1 min. The programme ended with a final extension at 72 °C during 10 min.

Samples found to be positive by Anaplasmataceae specific PCR were screened for *A. phagocytophilum* using forward and reverse primers -903f 5’-AGTTTGACTGGAACACACCTGATC-3’ and 1024r 5’-CTCGTAACCAATCTCAAGCTCAAC-3’ targeting a portion of the *msp2* gene (122 bp) [31]. The master mix was prepared as described above. Amplification started with an initial denaturation at 95 °C for 5 min, followed by 35 cycles of denaturation at 94 °C for 20 s, hybridisation at 50 °C for 30 s, elongation at 72 °C for 1 min and a final elongation at 72 °C for 10 min. A reference positive and negative sample were incorporated and amplified for each PCR.

PCR products were subsequently visualised through electrophoresis in 2% agarose gel using SYBR® Safe DNA gel stain (Invitrogen, Carlsbad, USA). The band size of the amplicon of interest was 650 bp.

### Purification, sequencing and phylogenetic analysis

Five positive PCR products of Anaplasmataceae spp. were selected for purification using the QIAquick PCR Purification Kit (Qiagen, Hilden, Germany). The purified DNA was then sequenced in both directions using the same primers as those used for PCR. Sequencing was performed by Inserm (Institut Cochin, 22, rue Mechain, 75 014 Paris, France; http://cochin.inserm.fr/les-plateformes/genomique-et-transcriptomique/plate-forme-de-sequencage/activite-sequencage).

GenBank BLAST analysis was used to compare the sequences obtained to those of reference strains. The algorithm ClustalW® and BioEdit® software were used to align multiple sequences. The dendrogram was constructed using the Mega software and the Neighbour joining (NJ) method for distance, parwise deletion and the boostrap test with 1,000 reiterations.

The software WinPepi® v11.42 [32] was used to compute the chi-square statistic.

## Results

39.62% (42/106) of camels showed a PCR band corresponding to the size of the nucleotide portion of the *groEL* target gene. The Oued Ed-Dahab region was found to have the highest percentage of positives, with 50.00% (9/18) of camels affected and the lowest rate in the region of Es-Semara, with only 12.50% (2/16). Using the Pearson chi-square test, the difference between the inter-regional prevalence was not found to be statistically significant (Chi-square 6 193, *P* > 0.05).

All four camels showing dependant oedema were found to be positive. Sampled camels were found to be exclusively infested with the tick species *Hyalomma dromedarii.* None of the 42 samples analysed using PCR targeting *msp2* gene was found to be positive for *A. phagocytophilum.* The results are summarised in Table 1.

**Table 1:** PCR (*groEL*) results for *Anaplasma* spp. across regions

Region	Number of samples	Number of positives- *Anaplasma* sp. (% pos)
Province of Tiznit	15	07 (46.67%)
Province of Guelmim	31	13 (41.94%)
Province of Laâyoune	14	06 (42.86%)
Province of Es-Semara	16	02 (12.50%)
Province of Boujdour	12	05 (41.67%)
Province of Oued Ed Dahab	18	09 (50.00%)
Total	106	42 (39.62%)

Out of the 42 Anaplasmataceae spp. PCR positive samples, one sample was randomly selected from each region giving a total of five samples sequenced with only one variant of *Anaplasma* sp. was found. The partial *groEL* sequences of *Anaplasma* sp. strains of the same lineage to “*Candidatus* Anaplasma camelii*”* isolated from camels were all 100% identical. One of these sequences was submitted to GenBank (GenBank accession number: KX074079).

GenBank BLAST analysis confirmed that these sequences were 100% genetically identical to “*Candidatus* Anaplasma camelii*”* [GenBank accession number: KJ814955] camel strains from Saudi Arabia [25]. Similarity with *A. platys* varied between 89% and 93% [GenBank accession number: AY008300, EU004824, EU004825, HQ718723, JN121382 and EU516386] and similarity with *A. phagocytophilum* between 83 and 84% [GenBank accession number: AY279085, JX133175].

**Fig. 3:**
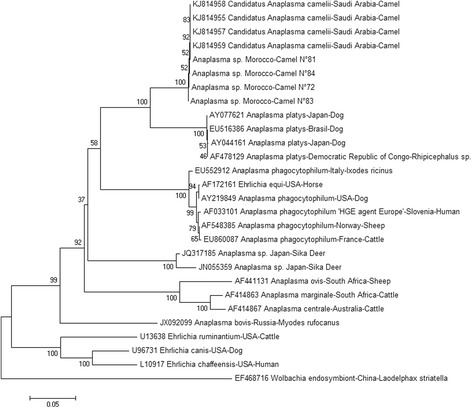
Phylogenetic tree of the *groEL* gene derived from Moroccan camel strains of *Anaplasma* sp. The tree was obtained using the neighbour joining method with software Mega after alignment with ClustalW of 650 bp sequences of the *groEL* gene from this study and *Anaplasma* sp. sequences available from GenBank from various host species and countries of origin. We used Kimura 2-parameter method to calculate distance matrices. In each node, percentages of bootstrap values (1 000 repeats) are indicated

Phylogenetic analysis of the 650 bp portion of *groEL* was undertaken for GenBank reference strains and study strains. Study sequences were found to cluster with those of “*Candidatus* Anaplasma camelii” [GenBank accession number: KJ814955, KJ814957-KJ814959] and to have a closer genetic lineage to *A. platys* than any other *Anaplasma* species. The high weight index of nodes obtained using the bootstrap-test as well as the identical amino acid sequences confirm this result (Fig. 3).

## Discussion

*Camelidae* are described as ‘pseudo’ ruminants and are distributed mostly in semi-arid and arid zones of Africa, Middle East and Asia. They are recognised for their ability to resist and thrive in the extreme and unfavourable conditions typical of these arid areas. Camel keeping is an important livelihood for populations of the southeast and southwest regions of Morocco providing income and provision of milk and meat. They are also used to transport nomads and their belongings during transhumance as well as traction.

There is a dearth of evidence on tick-borne (and other) diseases in camels, primarily because camels are kept by poor marginalised nomadic populations. Camel health is of limited interest to pharmaceutical companies because of perceived low profit-margins of drug sales and veterinary interventions. On a global scale, there are only a few studies on this group of pathogens in this species. This is in part due to the difficulty in accessing nomadic camel-keeping populations, by virtue of the fact that camels are few in number and dispersed across the immense area of the Sahara Desert. This is the first study on anaplasmosis in camels in the Kingdom of Morocco.

Anaplasmataceae spp. was found in 35.85% of ‘apparently healthy’ camels not showing clinical signs at the time of sampling; four (9.5%) of the 42 PCR-positive animals had clinical signs of infection. Camels sampled in this study were shown to be infected by a single variant of *Anaplasma* sp. identical to “*Candidatus* Anaplasma camelii” identified and named by Bastos et al. [25] for the same host species in Saudi Arabia. The related strain *A. platys* is known to cause cyclic thrombocytopenia in dogs. However, the pathogenicity of “*Candidatus* Anaplasma camelii” in camels and other species and its zoonotic potential are unknown. Nevertheless, it is important to note that sampling coincided with an outbreak of undiagnosed disease in 2013 onwards in Moroccan camels.

The four camels showing clinical signs of the previously undiagnosed disease came from different regions (Provinces of Laâyoune, Es-Semara, Boujdour and Oued Ed Dahab). The initial clinical sign observed was limited to dependent oedema that spread to the whole body, at which point the animals became recumbent and eventually died. In the absence of more in-depth diagnostic investigations, and given that trypanosomiasis is probably endemic in these regions [33, 34], the cause of this syndrome remains uncertain. As all four camels showing clinical signs were found to be PCR positive, we can suggest but not confirm that “*Candidatus* Anaplasma camelii” infection would have contributed in the clinical signs observed. Out of the five positive samples randomly selected and sequenced, only one was from the group of four camels showing clinical signs i.e. four of the sequenced samples were from apparently healthy camels; of the other three camels showing clinical signs all were positive for *Anaplamataceae* spp. but identification to species level will require further sequencing. Intra-erythrocytic anaplasmosis due to *A. marginale* is the only *Anaplasma* species confirmed to cause sub-clinical disease in camels [35].

Several studies have used the *groEL* gene ton discriminate between *Anaplasma* species [25, 30, 36, 37]. Sequencing of the *groEL* gene differentiated *Anaplasma* sp. variants circulating in camels and *A. platys* in dogs - which formed a cluster - and all other forms of anaplasmosis in different animal species - which formed a separate cluster (Fig. 3). Confirmation of infection with “*Candidatus* Anaplasma camelii” is limited to the five strains sequenced because (i) the *groEL* primers used to screen the samples, whilst preferentially amplifying *Anaplasma* species, are not specific to *Anaplasma* and (ii) without sequencing all amplicons one cannot conclude that all PCR positive samples contain “*Candidatus* Anaplasma camelii”. In a previous study which made use of the same primers [25], the presence of an *Ehrlichia* strain (closely related to *E. canis*) was detected in 3% of the dromedary camels from Saudi Arabia. As just five amplicons were selected for sequencing in this study of camels from Morocco, it is possible that *Ehrlichia* strains may also be present in camels from Morocco, and/or that multiple *Anaplasma* species may be present.

This study has shown that camels in Morocco are probably a reservoir for “*Candidatus* Anaplasma camelii” vectored by the tick *Hyalomma* spp. that becomes infected during its larval and nymphal stage by feeding on small desert animals. After maturation to the adult stage, the tick then inoculates the bacteria when taking a blood meal from larger mammals (e.g. camels, dogs and wild desert mammals such as the jackal).

Transmission to a range of domestic hosts is likely due to livestock management practices. As part of the transhumant lifestyle, camels, small ruminants and dogs belonging to the same owner are managed as a single unit. During the dry season, watering holes (known as *Guelta*) become severely limited and livestock keepers congregate with their livestock increasing risk of transmission as camel breeders in these areas rarely use preventive measures against ticks.

Prevalence values reported for other countries are lower than the 39.62% obtained here. A study by Bastos et al. [25] undertaken in Saudi Arabia using PCR targetting 16 s rRNA genes and the *groEL* gene reported a camel anaplasmosis prevalence of 26%. In this study no camels were found to be positive for *A. phagocytophilum*. However in Tunisia, a neighbouring country with similar camels rearing conditions and practices to those of Morocco, 29.2% of camels were seropositive for *A. phagocytophilum* [28]. This has to be interpreted with caution as cross-reactions with other *Anaplasma* species can occur [38, 39]. A later study re-screened blood samples from the same animals by PCR (16S rRNA) and prevalence was 17.7%. The strain in these Tunisian camels was characterised as *Anaplasma* sp. and considered as *A. platys*-like [26]. In the Canary Islands, anti *Anaplasma* sp. antibodies were detected in 3% of camels sampled [40]. In China, *A. platys* infections have been reported in clinically healthy Bactrian camels with 7.2% prevalence [27]. In Nigeria, prevalence ranged from 3.8 to 16.5% for intra-erythrocytic anaplasmosis due to *A. marginale* in camels [41–44]. However, infection of camels with *A. marginale* has never been confirmed using molecular methods, either in Nigeria or elsewhere in the world.

**Fig. 4:**
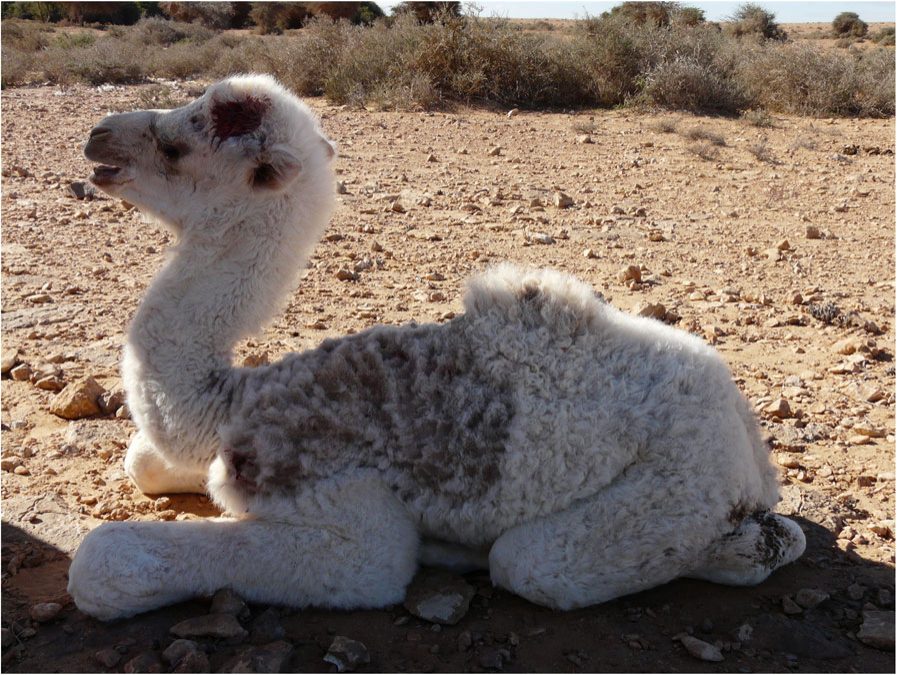
Mass tick infestation in a juvenile camel. Pruritus promotes hair loss in the thoracic region and head wounds through excessive rubbing

**Fig. 5:**
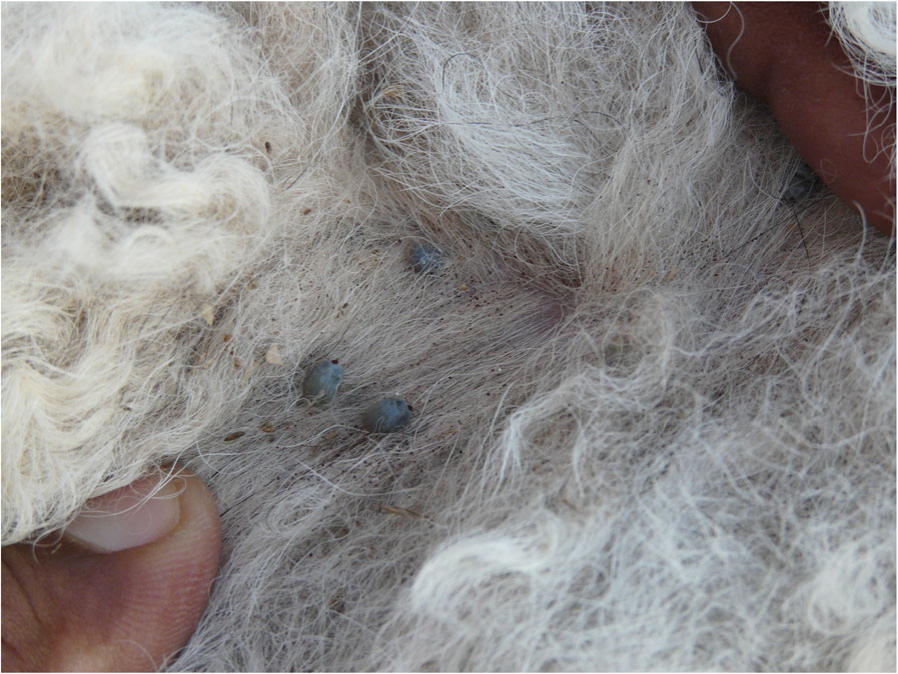
Mass tick infestation in a juvenile camel

Moreover, In agreement to what has been reported in Tunisia [26, 28], camels sampled in this study were mainly infested with hard ticks of *Hyalomma* sp. genus and not *Ixodes ricinus*. Heavy tick infestations were mostly observed in juvenile animals, which are infested with nymphs, referred to colloquially as *Delma*. This triggers intense pruritus and hair loss (Figs. 4 and 5), anaemia through mass tick feeding and eventually death if infected with a tick-borne disease.

In the Maghreb region, the tick genus *Hyalomma* sp. has been reported to be a potential vector of several pathogens, including *Rickettsia aeschlimannii*, *R. africae* [45–47], *Ehrlichia* sp. and *A. phagocytophilum* [48]. This tick is widely distributed in southern Morocco [49] as it is adapted to the extreme desert conditions, and it has been demonstrated to infest a large range of animals [50, 51], suggesting that *Hyalomma* sp. could also be a vector for “*Candidatus* Anaplasma camelii”.

## Conclusions

This is the first report of camel anaplasmosis in Morocco and to genetically characterise “*Candidatus* Anaplasma camelii”*.* Infection of camels with this bacterium does not seem to cause clinical disease, but the high prevalence would suggest that camels are the principal host of this pathogen. Further studies are required to determine: (i) the role of different vectors (tick and insect) in transmission (ii) the role of domestic and wild species as reservoir or dead-end hosts (iii) the zoonotic potential of this pathogen and (iv) its pathogenicity in camels.

## Additional file

Additional file 1: Multilingual abstracts in the six official working languages of the United Nations. (PDF 722 kb)

## Abbreviations

A: *Anaplasma*
DNA: Deoxyribonucleic acid
EDTA: Ethylenediaminetetraacetic acid
GA: Granulocytic anaplasmosis
HGA: Human granulocytic anaplasmosis
PCR: Polymerase chain reaction
rRNA: Ribosomal ribonucleic acid
TBF: Tick-borne fever
