# Supplementary material for: “Candidatus anaplasma camelii” in one-humped camels (Camelus dromedarius) in Morocco: a novel and emerging anaplasma species?
Source: Infect Dis Poverty. 2017 Feb 5;6:1. doi: 10.1186/s40249-016-0216-8 (PMC5292149; doi:10.1186/s40249-016-0216-8)
Supplement: Additional file 1: — Multilingual abstracts in the six official working languages of the United Nations. (PDF 722 kb) [file 40249_2016_216_MOESM1_ESM.pdf]

Translation of the abstract into the six official working languages of the United Nations

**مرض " كانديداتوس أنابلازما كاميلي" في الجمال وحيدة السنام في المغرب: هل هناك أنواع جديدة من بكتيريا أنابلازما؟**

**هشام آيت لباشا، زياد زواغي، سعيد العلي، عبد الكبير رحليم، إليزابيث بيتيت، ماري جولي دوكوروتي، هنري جان بولويس، و رينو مايار**

**ملخص**
**خلفية عن الموضوع:** تزايد الاهتمام بمرض أنابلازموسيس (anaplasmosis) التي تصيب الإبل بسبب ظهورها الأخير في أجسام هذه الحيوانات والمخاوف لقدرته الحيوانية المنشأ. لذلك ما تزال أمراض أنابلاسموسيز في الإبل بعيدة عن الفهم تمامًا لأن معظم ملاك الجمال هم من المهمشين الفقراء وغالبًا من البدو الرحل التي أهُملت مصالحهم لحد كبير. وقد اعتمدت معظم دراسات أنابلاسموسيز للإبل على الفحص المجهري والأمصال للتشخيص في ثلاث دراسات فقط، جرت في تونس والسعودية والصين، واستخدمت التشخيص الجزيئي. يحدد العمل الحالي سلالات *أنابلازماسيا* في جسم الجمل وحيد السنم في المغرب باستخدام تفاعل البوليميراز المتسلسل أو PCR.
**الأسلوب:** تم أخذ عينات عشوائية للإبل (*ن* = 106) من 6 مناطق تمثل البيئات الزراعية المختلفة في جنوب المغرب. تم جمع وفحص الدم بشكل كامل باستخدام أساليب PCR لاستهداف الجين *groEL*. تم تحديد سلالات *أنابلازماسيا* عن طريق تحليل تسلسل جينات *groEL*.
**النتائج:** أثبتت الفحوص أن 39.62٪ (106/42) من الإبل تحمل فيروس *أنابلازماسيا*. كشف تحليل أداة بحث الانتظام الأساسي (BLAST) لبنك الجينات خمس عينات متسلسلة إيجابية، أن جميع سلالات كانت 100٪ مطابقة لـ"*Anaplasma Candidatus* camelii". أدى التحقيق في نشوء وتطور والتشخيص الوراثي للجزء المنتظم (650 bp) من جينات *groEL* تشابه عال مع *A. platys*.
**الخلاصة:** توضح هذه الدراسة تداول الأنواع مجهولة سابقًا من أنواع أنابلاسما في المغرب والقريبة وراثيًا من العامل المتسبب في أنابلازموسيس للكلاب ولكن الذي يعتقد أن جسم الإبل وحيد السنم هو منشأه الأساسي.

Translated from English version into Arabic by RAMI ALHAMES, through

TRANSLATORS WITHOUT BORDERS

**摩洛哥单峰驼(*Camelus dromedarius*)体内发现的“*Candidatus* Anaplasma camelii”：一种新出现的无形体属种类？**

Hicham Ait Lbacha, Zaid Zouagui, Said Alali, Abdelkbir Rhalem, Elisabeth Petit, Marie Julie Ducrotoy, Henri-Jean Boulouis and Renaud Maillard

**摘要**
**引言**：骆驼无形体病越来越受到人们关注，因为骆驼成为保虫宿主并有可能造成人兽共患病。目前，关于骆驼的无形体病的流行病学还知之甚少，原因是它属于被边缘化的季节性迁移放牧的群体，很大程度上处于被忽视的地位。大多数骆驼无形体病的研究都依赖于显微镜镜检和血清学诊断，而且到目前为止，只在突尼斯、沙特阿拉伯和中国的 3 项研究采用了分子诊断方法。本研究描述了采用 PCR 方法鉴定流行于摩洛哥单峰驼（*Camelus dromedarius*）的无形体科（Anaplasmataceae）菌株的特征。
**方法**：从 6 个地区随机抽样 106 只骆驼分别代表摩洛哥南部地区不同的农业生态区域。收集全血并采用 PCR 方法鉴定 *groEL* 基因，并通过分析 *groEL* 基因序列表征无形体科菌株。
**结果**：通过筛查共发现 39.62% (42/106)的骆驼感染无形体科菌。GenBank BLAST 分析 5 条阳性序列，显示与 *Candidatus* Anaplasma camelii 的序列一致性为 100%。系统发育学和遗传特征分析 650 bp 的 *groEL* 基因片段确认其与 *A. platys* 高度相似。
**结论**：本研究表明之前在摩洛哥未确认的无形体属（*Anaplasma*）种类在遗传学上与犬无形体病的病原较近，但单峰驼是其主要的保虫宿主。

Translated from English version into Chinese by Xin-Yu Feng, edited by Pin Yang

CHINESE CENTER FOR DISEASE CONTROL AND PREVENTION
NATIONAL INSTITUTE OF PARASITIC DISEASES

**«*Candidatus* Anaplasma camelii» chez les dromadaires (*Camelus dromedarius*) marocains: l'émergence d'une nouvelle espèce d'Anaplasma?**

Hicham Ait Lbacha, Zaid Zouagui, Said Alali, Abdelkbir Rhalem, Élisabeth Petit, Marie Julie Ducrotoy, Henri-Jean Boulouis et Renaud Maillard

**Résumé**

**Contexte:** La récente émergence de l'anaplasmose des chameaux et des dromadaires suscite l'intérêt mais aussi l'inquiétude en raison de son potentiel zoonotique. L'épidémiologie de l'anaplasmose des camélidés est mal connue, principalement parce que ces animaux sont utilisés par des populations marginalisées, pauvres et souvent nomades, dont les intérêts sont fréquemment négligés. La plupart des études de l'anaplasmose des camélidés utilisent la microscopie et la sérologie ; seules trois études, réalisées en Tunisie, en Arabie Saoudite et en Chine, ont fait appel au diagnostic moléculaire. La présente étude caractérise par RCP les souches d'Anaplasmataceae en circulation dans l'espèce-réservoir *Camelus dromedarius* au Maroc.
**Méthodes:** Des dromadaires (*n*=106) ont été prélevés de façon aléatoire dans 6 régions représentant différentes zones agro-écologiques du sud du Maroc. Leur sang entier a été recueilli et étudié par RCP en visant le gène *groEL*. Les souches d'Anaplasmataceae ont été caractérisées par analyse des séquences du gène *groEL*.
**Résultats:** Au total, 39,62% (42/106) des dromadaires testés étaient positifs pour des Anaplasmataceae spp. L'analyse par GenBank BLAST de cinq échantillons positifs séquencés a révélé que toutes les souches étaient identiques à 100 % à «*Candidatus* Anaplasma camelii». L'examen phylogénétique et la caractérisation génétique du segment aligné (650 pb) du gène *groEL* ont confirmé une forte similitude avec *A. platys*.
**Conclusion:** Cette étude prouve la circulation au Maroc d'une espèce non identifiée jusqu'ici du genre *Anaplasma*, génétiquement proche de l'agent de l'anaplasmose canine mais dont le principal réservoir semble être *Camelus dromedarius*.

Translated from English version into French by Suzanne Assenat, through

TRANSLATORS
WITHOUT BORDERS

**Одногорбые верблюды (*Camelus dromedarius*) в Морокко несут в себе анаплизму верблюда (*Candidatus* Anaplasma camelii): это новый типовой вид анаплазмы?**

Hicham Ait Lbacha, Zaid Zouagui, Said Alali, Abdelkbir Rhalem, Elisabeth Petit, Marie Julie Ducrotoy, Henri-Jean Boulouis and Renaud Maillard

**Реферат**

**Фон:** В связи с тем, что на днях анаплазма верблюда появилась в хозяин-резервуаре и имеет потенциал зооноза, таким образом, возрастающее внимание уделяется анаплазме верблюда. Обыкновенно по причине того, что верблюды принадлежают к числу сезонного перегона скота и в значительной степени были игнорированы верблюдов воздействия, поэтому эпидемиология анаплазмы верблюда не известна. Большинство исследования ложится в основу диагнозов под микроскопом и серологии. В настоящее время, только проходили 3 исследования в Тунисе, Саудовской Аравии и Китае и в то же время пользовались молекулярной диагностикой. Данная работа объясняет о характеристиках пород анаплазмы марокканских одногорбых верблюдов по методу PCR.
**Методы:** Случайно выбрали 106 верблюдов из 6 районов как представители различных агроэкологических районов южного Марокко. С помощью метода PCR аттестовать ген, собрав чистокровность, также через исследование по гену groEL описать характеристики парод анаплазмы.
**Результаты:** С помощью метода отсеивания открыть всего 39.62% (42/106) верблюдов, несущих в себе семейству Anaplasmataceae СПП. GenBank BLAST сделал анализ для 5 позитивных

секвенированных образецев и обнаружил что, "*Candidatus* Anaplasma camelii" и пароды 100% сходились. Большое сходство между геном groEL и A. Platys были утверждены филогенетическими исследованиями и анализом о генетической характеристике (650 bp).
**Заключение:** Данное исследование показано круговорот неидентифицированных видов рода анаплазмы в последнее время в Марокко генетически имеет сходства с возбудителями анаплазмоза собак, однако одногорбый верблюд считается хозяин-резервуаром.

Translated from English version into Russian by Hao-qi Zhang

**«*Candidatus* Anaplasma camelii» en dromedarios (*Camelus dromedarius*) en Marruecos: ¿se trata de una nueva especie emergente de Anaplasma?**

Hicham Ait Lbacha, Zaid Zouagui, Said Alali, Abdelkbir Rhalem, Elisabeth Petit, Marie Julie Ducrotoy, Henri-Jean Boulouis y Renaud Maillard

**Resumen**

**Antecedentes:** A raíz de la reciente aparición de anaplasmosis en dromedarios como especie reservorio, cada vez existe más interés y preocupación por su potencial zoonótico. Todavía se sabe poco sobre la epidemiología de la anaplasmosis en dromedarios, sobre todo porque los dromedarios pertenecen a poblaciones pobres y marginales, a menudo trashumantes, cuyos intereses suelen quedar mayoritariamente descuidados. La mayoría de los estudios sobre anaplasmosis en dromedarios se basa en el diagnóstico microscópico y serológico, y solamente tres estudios realizados en Túnez, Arabia Saudita y China han utilizado el diagnóstico molecular. El presente trabajo describe las cepas Anaplasmataceae *que circulan en el reservorio Camelus dromedarius* de Marruecos utilizando PCR.
**Métodos:** Se tomaron muestras aleatorias de dromedarios (*n*=106) de 6 regiones agroecológicas diferentes del sur de Marruecos. Se extrajo sangre y se examinó el gen *groEL* utilizando métodos de PCR. Las cepas Anaplasmataceae se caracterizaron con un análisis de secuencias del gen *groEL*.
**Resultados:** El 39,62 % (42/106) de los dromedarios examinados dieron positivo para Anaplasmataceae spp. El análisis con GenBank BLAST de cinco muestras de secuencia positivas reveló que todas las cepas eran 100 % idénticas a «*Candidatus* Anaplasma camelii». La investigación filogenética y la caracterización genética del segmento alineado (650 bp) del gen *groEL* confirmaron que existe un elevado parecido con *A. platys*.
**Conclusión:** Este estudio demuestra la circulación de una especie no identificada anteriormente del gen *Anaplasma* en Marruecos que es genéticamente similar al agente que causa la anaplasmosis canina, pero cuyo reservorio principal se cree que es el *Camelus dromedarius*.

Translated from English version into Spanish by Núria Llorens, through

TRANSLATORS
WITHOUT BORDERS
